# Review of Lightweight, High-Temperature Thermal Insulation Materials for Aerospace

**DOI:** 10.3390/ma18102383

**Published:** 2025-05-20

**Authors:** Qi Zhang, Hongyan Huang, Chaoshuai Lei, Yuanyuan Liu, Wenjing Li

**Affiliations:** Aerospace Institute of Advanced Material & Processing Technology, Beijing 100074, China; casic306zhangqi@163.com (Q.Z.); leichaoshuai@126.com (C.L.); liuyy07@163.com (Y.L.)

**Keywords:** lightweight high-temperature thermal insulation materials, flexible ceramic fiber felts, thermal insulation tiles, aerogels, multilayer insulations

## Abstract

Lightweight, high-temperature thermal insulation materials play a critical role in aerospace applications, where extreme temperature conditions necessitate lightweight, high-performance solutions. This paper explores advancements in lightweight, high-temperature insulation materials specifically designed for aerospace environments, focusing on innovative flexible ceramic fiber felts, thermal insulation tiles, nano-insulation materials (aerogels), and multilayer insulations (MLIs). These materials exhibit superior thermal resistance, low density, and durability under dynamic and harsh conditions. Key developments include the integration of nanostructures to enhance thermal conductivity control and improve mechanical stability. This paper also highlights applications in spacecraft thermal protection systems, providing insights into the challenges of future material design strategies. These advancements underscore the growing potential of thermal insulations to improve energy efficiency, safety, and performance in aerospace missions.

## 1. Introduction

Lightweight, high-temperature thermal insulation materials are a category of advanced materials specifically engineered to meet rigorous performance standards [1,2,3,4,5,6,7]. They effectively reduce heat transfer at elevated temperatures while maintaining minimal weight and spatial requirements, thereby facilitating efficient energy use and providing reliable thermal protection for equipment [8,9]. This ensures the stable and efficient operation of systems, rendering these materials essential in critical sectors such as aerospace, marine engineering, and nuclear energy [10,11,12,13]. Moreover, they represent a significant focus of global research in advanced material technologies.

Practical heat transfer involves solid conduction, gas conduction, convection, and radiation. To address these complex heat transfer mechanisms, high-porosity, lightweight, ceramic porous materials are widely utilized worldwide [14,15,16,17]. These materials are particularly effective in minimizing solid phase heat transfer, while their designs vary mainly in their capacity to reduce gas phase and radiative heat transfer. Notable examples include flexible ceramic fiber felts, thermal insulation tiles, nano-insulation materials (aerogels), and high-temperature multilayer insulation (MLI) systems. In this paper, based on the reported applications and developments of these materials in the aerospace field, the characteristics and research of these four materials will be introduced, followed by a discussion on the development prospects of lightweight materials, and an outlook on the development of lightweight, high-temperature thermal insulation materials for aerospace applications will be presented.

## 2. Flexible Ceramic Fiber Felts

Fiber insulation structures are widely used in flexible thermal protection systems [18,19,20], consisting of layered, stitched configurations of fiber insulation mats, fabrics, and threads [21,22,23,24]. These structures are primarily applied in low-load thermal regions of aerospace vehicles, such as upper wing surfaces, tail fin sections, elevator controls, and braking mechanisms, providing effective thermal management while maintaining structural flexibility and durability [25,26].

**Figure 1:**
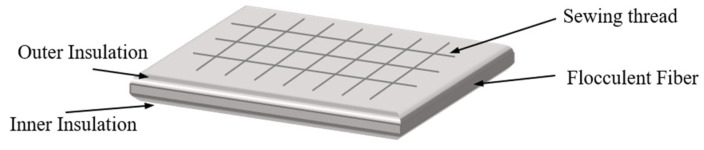
Structure of Flexible Reusable Surface Insulation.

During the initial development of flexible thermal protection systems, the United States pioneered the use of Nomex felt as a flexible thermal insulation material for aerospace applications, with an effective temperature range limited to below 370 °C. With subsequent advancements in technology, U.S. researchers developed the Flexible Reusable Surface Insulation (FRSI) structure [27] (Figure 1). This design utilized fused silica fibers as the primary material, featuring a construction in which silica fiber mats were encapsulated by silica fiber fabric and secured through stitching with silica fiber threads. Due to the exceptional thermal properties of silica fibers, the temperature resistance of aerospace vehicles was significantly enhanced, reaching up to 815 °C [28].

**Figure 2:**
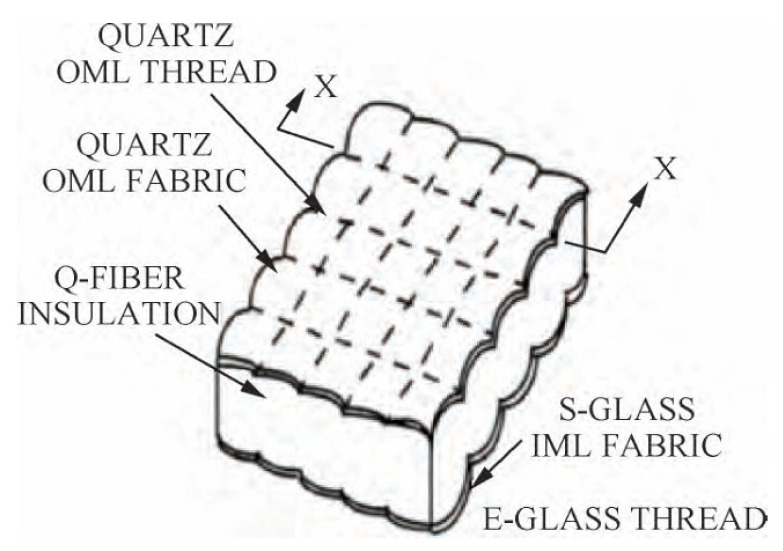
Structure of Advanced Flexible Reusable Surface Insulation [33].

The NASA Ames Research Center has introduced an innovative advancement by enhancing the design of Flexible Reusable Surface Insulation (FRSI). This improvement incorporates borosilicate aluminum-based fiber threads and fabrics, resulting in the development of Advanced Flexible Reusable Surface Insulation (AFRSI, as shown in Figure 2). The AFRSI structure exhibits an exceptionally low thermal conductivity of approximately 0.033 W·m^−1^·K^−1^ under standard temperature and pressure conditions, allowing aerospace vehicles to endure temperatures as high as 1037 °C [29]. Due to its superior thermal insulation performance, AFRSI has been widely utilized in the X-51A hypersonic vehicle, which achieved flight speeds of up to Mach 10 [30]. Building on this foundation, researchers subsequently developed two novel thermal protection materials: Carbon Fiber Blanket Insulation (CFBI) and Tailorable Advanced Blanket Insulation (TABI) [31,32]. CFBI, constructed using silicon carbide fiber threads and mats, and TABI, which employs borosilicate aluminum or silicon carbide fiber mats, further enhance the thermal resistance and operational temperature thresholds of aerospace vehicles.

**Figure 3:**
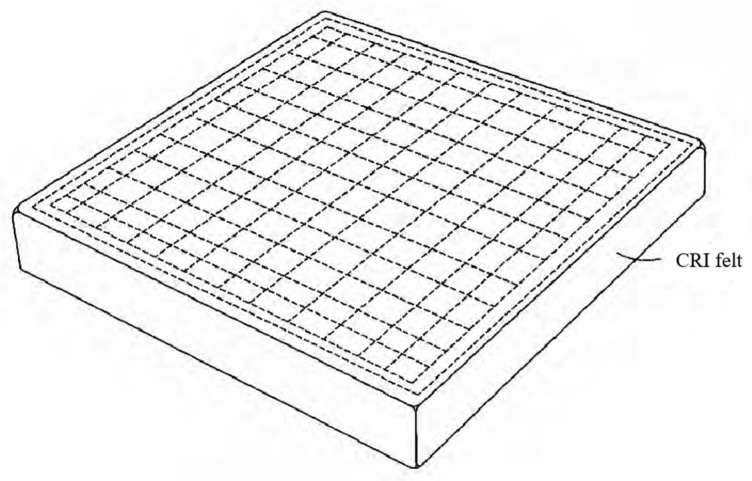
Structure of conformal reusable insulation [33].

In the 21st century, the United States made significant advancements in flexible thermal protection materials by developing a novel conformal reusable insulation (CRI, as shown in Figure 3) material [34]. This insulation is composed of ceramic fibers, including silica, alumina, and boron oxide, which form the intermediate fiber mats. To meet the specific environmental requirements of high- and low-temperature surfaces, borosilicate aluminum fiber fabric is applied to the high-temperature side, while quartz fiber fabric is utilized on the low-temperature side. Furthermore, building on existing flexible thermal protection materials, CRI incorporates a ceramic coating on the outermost layer, enhancing both its thermal and erosion resistance. As a result, the temperature tolerance of aerospace vehicles is increased to 1204 °C, and CRI has been successfully deployed on the windward surfaces of the X-37A and X-37B orbital test vehicles [35]. The parameters of some flexible ceramic fiber felts are provided in Table 1.

In addition to the United States, several other countries have initiated research on fiber insulation mat materials. The European aerospace and defense group Astrium has developed a flexible external insulation (FEI) suitable for spacecraft surfaces [36]. This material is created by sewing silica or glass fabrics, which offer a high radiation coefficient. Meanwhile, Nanjing University of Aeronautics and Astronautics has developed a composite insulation mat made of hollow microspheres as the matrix, with glass fibers as the primary component. The thermal insulation mechanism of this composite mat relies on infrared absorption, demonstrating high strength and excellent dimensional stability. Langbo New Materials Technology Co., Ltd. (Shanghai, China) has produced an oxidation-resistant carbon fiber insulation mat using chopped carbon fibers as the matrix. This insulation mat not only provides effective thermal insulation and oxidation resistance but also features strong fiber retention, minimizing the risk of fiber shedding.

**Table 1:** Parameters of the Flexible Ceramic Fiber Felts [31,37,38].

Materials	Density/(g·cm^3^)	Using Temperature Range/°C
AFRSI	0.128	650
TABI	Adjustable	800~1200
CFBI	0.174	~1300
CRI	~0.2	~1200

Overall, flexible fiber felts exhibit advantages such as low density, high flexibility, excellent high-temperature resistance, and low thermal conductivity, making them widely utilized in spacecraft thermal protection systems. However, limitations in mechanical strength and long-term stability restrict their applicability in extreme environments. Furthermore, with the continuous advancement of thermal protection materials, the thermal insulation performance of flexible fiber felts is gradually becoming insufficient for practical applications. Future research and development efforts should focus on material composition optimization, the application of nanomodification technologies, and multifunctional integrated design to further enhance thermal insulation performance, durability, and environmental adaptability, thereby meeting the stringent requirements of deep-space exploration and reusable spacecraft.

## 3. Thermal Insulation Tiles

**Figure 4:**
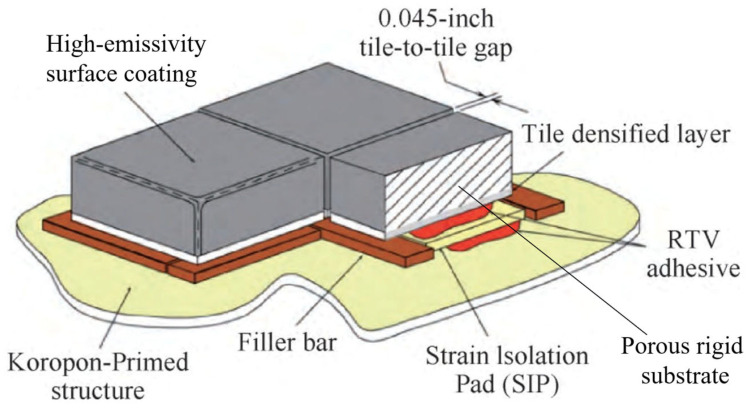
Typical structure of thermal insulation tiles [33].

Thermal insulation tiles are a crucial component of spacecraft thermal protection systems (TPSs), especially on the windward surfaces where heat shielding is most critical. These tiles fulfill their primary function by integrating two essential elements: a high-emissivity surface coating and a porous rigid substrate [39,40,41,42,43] (as shown in Figure 4). The high-emissivity surface coating, designed with a dense and robust structure, effectively radiates the majority of absorbed heat back into the surrounding environment while withstanding aerodynamic forces. Beneath this coating lies a porous rigid substrate, a microstructure formed by interwoven, high-temperature-resistant short fibers with a porosity exceeding 90%. This configuration imparts key advantages, such as lightweight construction, exceptional thermal resistance, and low thermal conductivity, making it an outstanding thermal insulation material. Over the past half-century, thermal insulation tiles have been widely used in the windward surfaces and other areas of various types of spacecraft.

**Figure 5:**
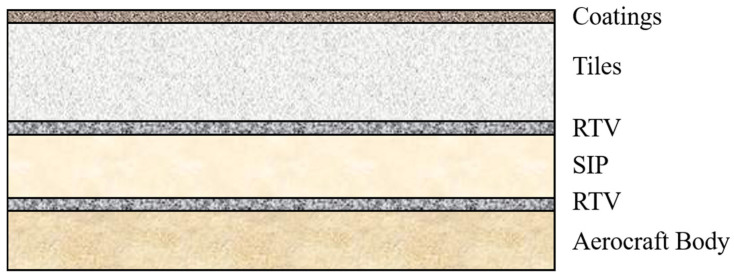
Connection method between the thermal protection tiles and the spacecraft.

Despite these advantages, the inherent rigidity and brittleness of the tiles render them susceptible to fractures resulting from deformation or impact [44]. Consequently, direct bonding to the metallic skin of spacecraft is not feasible. To address this limitation, strain isolation pads are employed for attachment, and precisely engineered gaps between the tiles accommodate deformation mismatches between the metallic skin and the external thermal protection layer, while also facilitating ventilation (as shown in Figure 5) [45].

**Figure 6:**
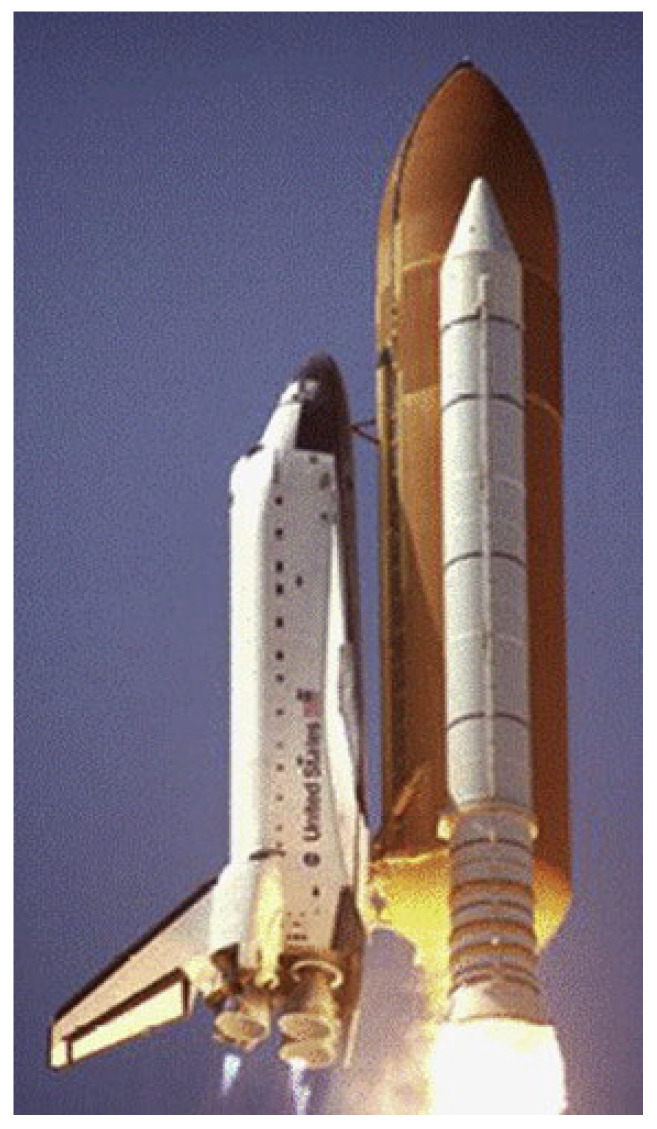
Columbia space shuttle [49].

The practicality of this design was first demonstrated in April 1981 during the maiden flight of the Columbia space shuttle (as shown in Figure 6), which featured over 24,000 insulation tiles [46]. This landmark event underscored the effectiveness of these tiles as the primary thermal protection system (TPS) for space shuttles. Over the following decades, insulation tiles continued to be the material of choice for large-scale thermal shielding on windward surfaces, contributing to the successful completion of more than 130 missions. The development of insulation tiles, initiated in the United States during the 1960s, has since established and maintained a leading global position in thermal protection technology [47,48].

The space shuttle program in the United States utilized five types of rigid ceramic tiles: LI-900, LI-2200, FRCI-12, AETB-8, and BRI-18, which can be broadly classified into three generations.

The first-generation thermal insulation tiles, LI-900 and LI-2200 [50], were developed by Lockheed in the 1970s and are composed of pure quartz fibers. Among these, LI-900 became the most widely utilized material in the space shuttle program due to its lightweight and low thermal conductivity. In contrast, LI-2200, which shares a similar composition with LI-900, was primarily employed in areas requiring greater strength, such as the forward windows and cabin doors, although it had the disadvantage of a higher density. Additionally, the poor compatibility between the pure quartz substrate of the LI series tiles and the later-developed TUF-1 impact-resistant coating led to unresolved impact resistance issues. This challenge significantly restricted the application of these thermal insulation tiles in the external thermal protection systems of subsequent reusable spacecraft.

FRCI-12 [51], a second-generation ceramic thermal insulation tile, is composed of a binary system of quartz fibers and borosilicate aluminum fibers, which have a higher softening temperature and sintering temperature. Developed by NASA’s Ames Research Center in the 1980s, it was primarily designed to address the high-density issue found in LI-2200. FRCI-12 exhibits a significant increase in maximum operating temperature, raising it by nearly 100 °C. However, its thermal shock resistance is lower than that of the pure quartz fiber-based LI tiles, and its thermal conductivity is higher than that of LI-900, which somewhat limits its suitability for large-scale applications.

AETB-8 tiles [52], developed by NASA’s Ames Research Center in the 1990s, represent a third generation of rigid thermal insulation tiles, serving as an improved version of the FRCI tiles. These tiles are composed of borosilicate aluminum fibers with smaller diameters, which results in a more uniform fiber distribution during the manufacturing process. This refinement significantly enhances both the strength and thermal resistance of the tiles. Additionally, AETB tiles incorporate alumina fibers to partially replace the borosilicate aluminum fibers, further improving their temperature resistance. However, this enhancement is accompanied by a slight reduction in the mechanical strength of the tiles.

In addition to the three previously mentioned thermal insulation tiles, Boeing developed the BRI-18 tile in the early 2000s [53]. This tile features a binary fiber system composed of 60% to 80% quartz fibers and 20% to 40% alumina fibers. It includes 0.1% to 1% boron-containing powder as a sintering agent, along with a small quantity of silicon carbide powder to enhance radiation resistance. Compared to the AETB tiles developed by NASA’s Ames Research Center, the BRI-18 demonstrates lower thermal conductivity. The performance parameters of some thermal insulation tiles are provided in Table 2.

Rigid thermal insulation tiles provide numerous advantages, including lightweight construction, high-temperature resistance, exceptional thermal insulation properties, and reusability. However, they also present significant challenges, such as brittleness, complex assembly processes, prolonged maintenance and repair cycles, high costs, and a propensity to crack and detach due to ice formation after water absorption. These issues resulted in considerable operational difficulties during the later stages of space shuttle missions. Specifically, each maintenance cycle of the thermal protection system demanded nearly 40,000 man-hours, which significantly extended launch intervals and diminished mission efficiency.

Overall, thermal insulation tiles exhibit excellent high-temperature resistance, low thermal conductivity, and strong ablation resistance, making them a key material for spacecraft reentry thermal protection systems. However, their high brittleness, limited impact resistance, and high manufacturing and maintenance costs constrain their efficiency in reusable spacecraft applications. Future development efforts should focus on the research and development of high-toughness ceramic matrix composites, the integration of nanostructured thermal insulation materials, and the application of intelligent self-healing technologies to enhance mechanical performance, service life, and adaptability to complex environments, thereby meeting the demands of next-generation spacecraft.

## 4. Aerogels

**Figure 7:**
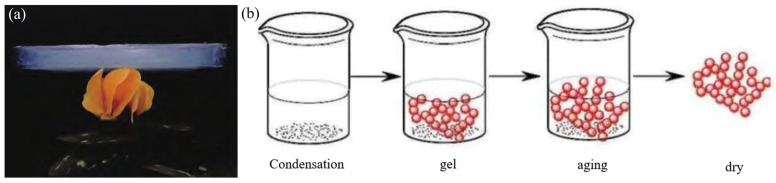
(**a**) Aerogels. (**b**) Preparation process of aerogels [64].

Aerogels are highly dispersed solid materials characterized by a nanoporous network structure composed of nanometer-scale colloidal particles, with a gaseous medium filling the pores (as shown in Figure 7a) [57,58,59,60,61]. Aerogel materials possess characteristics such as extremely low density, ultra-low thermal conductivity, high specific surface area, and high porosity, which have led to their widespread application in the aerospace field in recent years. First synthesized in the 1930s by Kistler [62], aerogels were produced using water glass through a sol-gel process combined with supercritical drying techniques. Kistler recognized their potential for applications such as catalysis and thermal insulation. However, technological constraints at the time hindered significant progress in aerogel research for several decades. A breakthrough occurred in the 1970s when Stanislaus Teichner [63] and collaborators, while investigating porous materials for oxygen storage and rocket fuel applications, replaced sodium silicate with tetramethyl orthosilicate (TMOS). By hydrolyzing TMOS in methanol to produce alcogels, they successfully fabricated SiO_2_ aerogels through controlled drying processes (as shown in Figure 7b). This innovation marked the beginning of rapid advancements in aerogel science and manufacturing.

**Figure 8:**
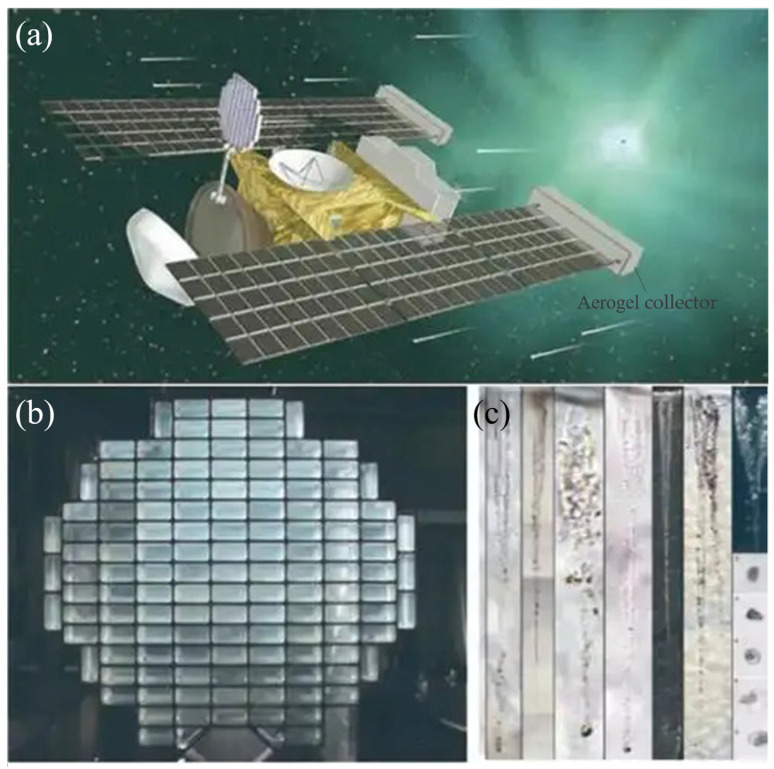
(**a**) Concept illustration of the Stardust mission. (**b**) Aerogel collector for comet dust. (**c**) Trajectory diagram of the comet dust captured by the aerogel [64].

In the past two decades, research into aerogels has accelerated, resulting in significant improvements in fabrication methods, material properties, and potential applications. SiO_2_ aerogels, in particular, have become a focal point due to their exceptional thermal, optical, and mechanical properties [65,66,67]. Notable applications include their use by the Swedish company Airglass as a medium in Cherenkov detectors for measuring the mass and energy of high-energy particles [68]. Similarly, NASA has utilized SiO_2_ aerogels in space shuttle missions to capture and analyze high-velocity cosmic dust from comets and interstellar space (as shown in Figure 8) [69]. These advancements highlight the growing versatility and importance of aerogels in both scientific and industrial fields.

Low-density SiO_2_ aerogel-based thermal insulation composites are increasingly recognized for their ability to provide thermal protection comparable to traditional materials while significantly reducing weight and volume. This unique advantage is particularly critical for thermal protection systems in aerospace applications [70,71,72,73,74,75]. Among global advancements, fiber-reinforced SiO_2_ aerogel composites developed by the U.S. company ASPEN stand out, exhibiting exceptional performance with thermal conductivities ranging from 0.013 to 0.016 W/m·K at room temperature and 0.033 W/m·K at 500 °C. Compared to conventional inorganic insulation materials, SiO_2_ aerogels demonstrate superior thermal insulation efficiency and a slower rate of increase in thermal conductivity with temperature. Additionally, unlike organic insulation materials, SiO_2_ aerogels offer outstanding high-temperature resistance, further solidifying their role in aerospace and military applications.

**Figure 9:**
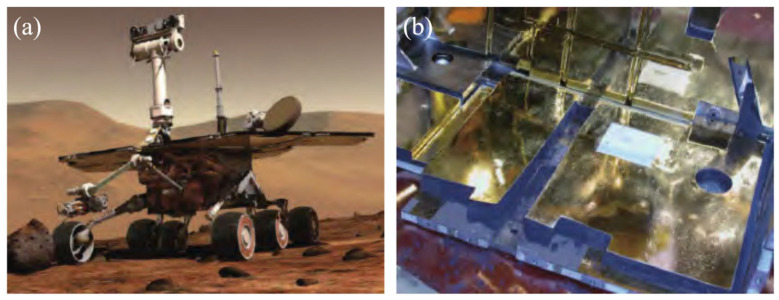
(**a**) Concept illustration of the NASA Mars rover. (**b**) Aerogel thermal insulation material for Mars rovers [64].

NASA has successfully demonstrated the potential of these materials in space exploration. For instance, SiO_2_ aerogel composites were utilized as insulation layers in the Mars Rover, allowing the spacecraft to withstand extreme low temperatures below −100 °C (as shown in Figure 9) [76,77]. At the NASA Ames Research Center, aluminum silicate fiber-reinforced SiO_2_ aerogels were developed for use in space shuttles. These composites feature a refractory aluminum silicate fiber framework filled with nanoporous aerogel, resulting in improved thermal insulation performance and reduced thermal conductivity compared to traditional refractory materials.

China has made significant advancements in the research and application of aerogel materials. SiO_2_ aerogels have been utilized as thermal protection materials in prominent spacecraft, including Tianwen-1 and Long March 5. In terms of research, the National University of Defense Technology has developed silica aerogel composites by integrating ceramic fibers with silica sol and utilizing supercritical drying techniques. These composites demonstrated thermal conductivities of 0.017 W/(m·K) at 200 °C and 0.042 W/(m·K) at 800 °C, along with mechanical properties such as compressive strength (0.98 MPa at 10% strain), tensile strength (1.44 MPa), and flexural strength (1.31 MPa) [78].

Despite these advancements, studies have identified a significant limitation: SiO_2_ aerogels experience considerable sintering and pore structure collapse at temperatures ranging from 600 °C to 1000 °C, which severely compromises their thermal insulation performance [79,80,81]. This limitation restricts their use in environments exceeding 1000 °C, presenting a challenge that requires further research and innovation to fully realize the potential of these materials for high-temperature applications.

To enhance the thermal stability of silica aerogels, researchers have incorporated alumina into the silica matrix, resulting in alumina–silica aerogels that exhibit improved thermal resistance and can withstand temperatures exceeding 1200 °C [82]. Studies indicate that these alumina–silica aerogels maintain a high specific surface area even after heat treatment at temperatures ranging from 1100 °C to 1300 °C. For instance, Yu et al. [83] fabricated a porous framework by sintering chopped quartz fibers with a binder, followed by impregnation with alumina–silica sol. After ambient pressure drying, the resulting alumina–silica aerogel composite demonstrated a thermal conductivity of 0.049 W/(m·K) at room temperature. Following heat treatment at 1100 °C, the thermal conductivity increased slightly to 0.057 W/(m·K), with no significant shrinkage observed along the length of the material.

Similarly, Li et al. [84] developed alumina–silica aerogel composites by utilizing a carbon fiber porous framework as a reinforcing phase. These composites exhibited a density of 0.376 g/cm^3^ and achieved a compressive strength of 5.44 MPa. In an argon atmosphere, the thermal conductivities were measured at 0.081 W/(m·K) at room temperature and 0.330 W/(m·K) at 1000 °C. In contrast, Xu et al. [85] employed a different method by impregnating mullite fibers with a polycarbosilane solution, followed by high-temperature pyrolysis to create a silicon carbide (SiC) coating on the fibers. This SiC coating significantly enhanced the extinction coefficient of the fibers in the 2.5–7.5 μm wavelength range, leading to a further reduction in the high-temperature thermal conductivity of the composites. At 1000 °C, the thermal conductivity was as low as 0.049 W/(m·K).

While alumina–silica aerogels demonstrate improved thermal stability, they still experience sintering at temperatures exceeding 1200 °C. Furthermore, their thermal conductivity at high temperatures increases with rising temperatures, which restricts their use in extreme environments. Currently, there are no documented applications of these aerogels in the aerospace industry, underscoring the necessity for further research to address these challenges and enhance their performance at elevated temperatures.

In summary, aerogel materials possess extremely low thermal conductivity and low density, making them highly promising for spacecraft thermal protection and insulation applications. However, their high production costs, and degradation in thermal stability and insulation performance at elevated temperatures limit their widespread application. Future research efforts should focus on enhancing their high-temperature stability, thermal insulation capability, environmental adaptability, and engineering reliability to meet the increasingly stringent requirements of deep space exploration, crewed space missions, and reusable spacecraft.

## 5. Multilayer Insulation System

**Figure 10:**
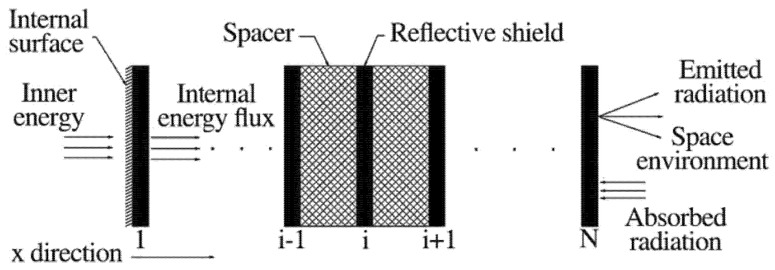
Schematic diagram of MLI [91].

The concept of multilayer insulation (MLI, as shown in Figure 10) was introduced by Swedish researchers in 1951 [86]. In terms of heat transfer mechanisms, when solid conduction and heat transfer through gaseous media are minimized to a certain extent, radiation becomes the predominant mode of heat transfer. Consequently, the combination of thin films with high-reflectivity and low-thermal-conductivity spacer materials results in effective thermal insulation. The thermal conductivity of these materials in a vacuum at room temperature typically falls within the range of 10^−3^ to 10^−4^ W/(m·K) [87,88,89]. Over the years, MLI materials have been extensively applied in aerospace, space energy, and various other industries worldwide. Depending on their operating conditions, these materials can be categorized into low-temperature and high-temperature MLI materials.

**Figure 11:**
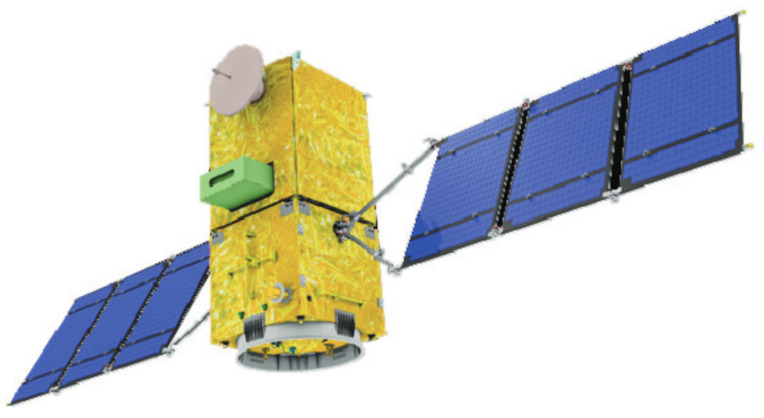
MLI on the satellite surface [92].

Low-temperature multilayer insulation (MLI) materials are typically utilized at temperatures below 300 °C. In these relatively mild environments, these materials often consist of polyester or polyimide films coated with gold or aluminum, combined with low-density spacer layers such as loose fibers, fabrics, or mesh. The application of thin metal coatings on low-density films, which weigh significantly less than pure metal foils of the same thickness, enables the achievement of both lightweight construction and high thermal insulation efficiency. These materials have been widely adopted in deep space satellites (as shown in Figure 11), cryogenic storage tanks, and various other applications, with thermal conductivities in a vacuum at room temperature reaching values around 10^−4^ W/(m·K) [90].

In contrast, high-temperature multilayer insulation (MLI) materials, designed for use at temperatures exceeding 600 °C, have developed at a slower pace. As the operating temperature rises, gold-coated metal films become unsuitable due to their limited thermal stability, making high-temperature-resistant pure metal foils the preferred choice. However, this preference results in a significant increase in weight. Additionally, at elevated temperatures, the emissivity of metal surfaces increases substantially, leading to a greater number of metal foil layers required, which further contributes to the overall weight of the material. While low-temperature MLI structures remain stable and maintain excellent insulation properties in cold environments, high temperatures introduce several challenges. Even pure metal foils, when subjected to prolonged exposure to high temperatures, may experience softening, creep, or other forms of thermal degradation. These issues can lead to interlayer metal bonding or high-temperature diffusion and melting, resulting in and a marked reduction in insulation performance. Therefore, compared to low-temperature MLI materials, high-temperature multilayer insulation presents greater challenges and complexities in terms of material design for effective high-temperature thermal protection.

Currently, high-temperature multilayer insulation (MLI) materials are most commonly used in applications such as space nuclear power systems, thermal protection for hypersonic vehicles, and engine insulation.

Space nuclear power systems are essential for deep space exploration, particularly in areas such as the Moon, Mars, and asteroids, where solar energy is inadequate. These systems are recognized for their stability, long-term operation, and reliable energy supply. The fundamental principle of these systems is to capture the substantial heat produced by nuclear reactions and convert it into electrical or mechanical energy. To optimize the utilization of heat released by the high-temperature reactor core, insulation materials are utilized to regulate heat flow and minimize thermal losses. Given the significant advantages of multilayer insulation (MLI) structures in high-vacuum environments, high-temperature MLI materials are typically the preferred choice for these applications.

**Figure 12:**
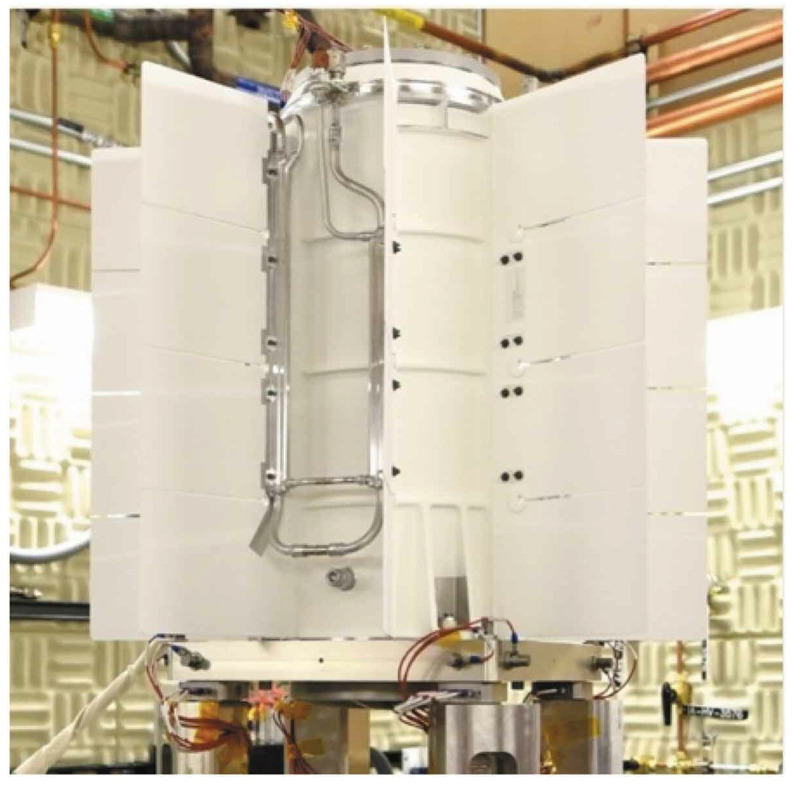
Radioisotope thermoelectric generator [94].

In the field of space nuclear power technology, the most advanced application is the use of radioisotope thermoelectric generators (RTGs, as shown in Figure 12) developed by the United States. By 2020, nearly 50 RTG systems had powered approximately 27 spacecraft, including Voyager 1 and 2, Galileo, Curiosity, and Opportunity, contributing to the development of a general-purpose RTG. The RTG systems used in the Voyager 1/2 and Galileo missions exemplify effective thermal insulation materials, utilizing molybdenum/quartz fiber cloth products [93]. This solution utilizes molybdenum foil as the reflective layer and quartz fiber cloth as the spacer layer for high-temperature multilayer insulation. The system comprises 60 layers of insulation, with a total thickness of approximately 18 mm and a bulk density of about 0.52 g/cm^3^. The molybdenum foil has a thickness of approximately 8 microns and is custom-manufactured by local suppliers, approaching the limits of current fabrication processes. This thickness enables it to endure long-term operating temperatures of around 1700 °C. The quartz fiber cloth, with a thickness ranging from 50 to 120 microns, is sourced from JPS Composite Materials’ Astroquartz series, which has a long-term temperature tolerance of approximately 1050 °C, thereby meeting the requirements for environments up to 1000 °C. Testing has demonstrated that the thermal conductivity of this material in a high-vacuum environment at 1000 °C is as low as 10^−2^ to 10^−3^ W/(m·K).

This material solution has been extensively utilized in practical applications, demonstrating exceptional reliability and stability. For example, the radioisotope thermoelectric generator (RTG) system in the Voyager spacecraft has been operational for nearly 47 years and continues to provide power, further validating the robustness of the design. Additionally, this solution is adaptable for higher operating temperatures. The molybdenum foil remains stable at temperatures up to 1700 °C, and the spacer layer, in addition to quartz fiber products, can be replaced with zirconia fiber products to enhance thermal resistance, thereby allowing for further increases in temperature.

However, the high surface density of 9.8 kg/m^2^ in this multilayer insulation solution poses challenges for the development of high-power space nuclear batteries. The equal surface density of 50% for both the metal foil and spacer layer offers significant opportunities for optimization, which has been a primary focus for research institutions such as NASA’s Glenn Research Center and the Jet Propulsion Laboratory (JPL).

Under the auspices of the large-scale dynamic nuclear power project SP-100, Thermo Fisher Scientific has conducted over a decade of research and validation, significantly reducing the weight of the spacer layer by employing micron-sized oxide particle coatings (e.g., zirconia, thorium oxide, yttria) to replace the quartz fiber spacer in general-purpose radioisotope thermoelectric generators (RTGs) [95]. This method involves spraying a mixture of adhesive and oxide particles onto a metal foil, resulting in a spacer layer approximately 10 microns thick, which weighs only about 1% of the corresponding foil material. This approach effectively achieves both weight reduction and enhanced insulation.

Building on this, Thermo Fisher conducted high-temperature stability assessments of various metals and their interactions with zirconia, testing for durations of up to 1000 h. The results indicated that zirconia remained stable at 1000 °C with metals such as molybdenum (Mo), nickel (Ni), and copper (Cu), with the exception of a reaction with niobium (Nb) near 1000 °C. In the absence of an oxide spacer, Ni-Ni bonding occurred at 600 °C; however, when zirconia was present, Ni foils in close proximity began to melt around 1100 °C. Significant diffusion defects, such as voids caused by the Kirkendall effect, were observed at the Ni-Mo and Ni-Nb interfaces. Additionally, metals like Ni and Nb, when in contact with Cu, rapidly formed low-melting-point alloys. These findings suggest that the lack of a high-porosity spacer layer can lead to increased risks associated with metal interlayer bonding. Therefore, ensuring the reliability of oxide–metal adhesion, particularly under conditions such as vibration or pressure detachment during flight, is critical. Since the termination of the SP-100 project in the late 20th century, further research on weight reduction design and reliability verification has not been reported [96].

Following these findings, NASA’s Jet Propulsion Laboratory (JPL) conducted extensive research on the selection of reflective layers as part of the SP-100 project. Beginning with 8-micron molybdenum foil as the baseline, JPL proposed several types of foil that are suitable for high-temperature multilayer insulation materials in space nuclear power systems. Among these, foils made from carbon, niobium, titanium, and zirconium demonstrated significant advantages in terms of weight, temperature resistance, and stability, showing the potential for weight optimization ranging from 44% to 85% compared to the 8-micron molybdenum foil [97].

Significant research has been conducted globally on high-temperature multilayer insulation materials for large-area spacecraft and power systems. The NASA Ames Research Center developed a new flexible multilayer thermal protection structure, featuring borosilicate aluminum fabric on both sides and insulation layers composed of silica felt, borosilicate aluminum, and alumina fiber ceramic materials. This structure incorporates ten layers of 10–20 micron stainless steel foil as the reflective shield, with borosilicate aluminum fine yarn cloth serving as the spacer layer [98]. Thermal coupling calculations indicate that this multilayer structure significantly outperforms flexible fiber insulation materials, such as AFRSI and AETB ceramic tiles, in terms of thermal conductivity and response, resulting in a substantial optimization of surface density. Its operational temperature range is between 500 °C and 1000 °C. This structure has been further developed with support from the European Space Agency, which replaced mid- to low-temperature fiber-based spacer layers with Aspen’s Pyrogel aerogel spacer, greatly enhancing the material’s thermal insulation properties and positioning it as the primary material solution for next-generation aerospace vehicle thermal protection systems [95].

In conclusion, while high-temperature multilayer insulation materials with exceptional temperature resistance and insulating properties have been successfully applied, the extensive use of pure metal foils and relatively high-density, reliable spacer layers significantly increases the overall weight of the materials. The challenge lies in designing lighter, more temperature-resistant, and reliable material systems through the optimization of foils, spacer layers, and lamination structures. This remains a critical area of focus in the development of high-temperature multilayer insulation materials. Additionally, high-temperature multilayer insulation materials may experience issues such as interlayer delamination, limited impact resistance, and complex manufacturing processes in extreme environments, which affect their long-term stability and reliability. Future development efforts should focus on the introduction of novel high-temperature reflective layer materials, optimization of interlayer structures, and the application of intelligent adaptive thermal insulation technologies to enhance their thermal protection performance, mechanical strength, and engineering adaptability, thereby meeting the increasingly stringent requirements of aerospace missions.

## 6. Development and Prospect

High-temperature, lightweight thermal insulation materials for aerospace applications are rapidly evolving, with a dual focus on reducing weight and enhancing thermal insulation efficiency. As the operational environment of spacecraft becomes increasingly complex, the requirements for insulation materials extend beyond mere high-temperature resistance. There is now an emphasis on providing exceptional thermal protection under extreme conditions while minimizing the weight of the materials. These lightweight, high-efficiency insulating materials directly impact fuel consumption, payload capacity, and mission reliability, making them critical components for the future development of aerospace technology.

Weight reduction is a primary objective in the development of aerospace insulation materials. By decreasing the density of the insulation layer, the overall mass of the spacecraft can be significantly reduced, thereby improving the efficiency of the propulsion system and creating additional capacity for scientific instruments or fuel. Furthermore, lightweight materials simplify the structural design of spacecraft, contributing to lower manufacturing and maintenance costs. However, it is crucial that the pursuit of lighter materials does not compromise thermal performance. Efficient thermal insulation remains fundamental to the success of space missions. Consequently, new insulation materials are being optimized for ultra-low density and minimal thermal conductivity, thereby enhancing thermal protection while preserving structural integrity.

The development of multifunctional composite materials and the application of advanced manufacturing technologies are emerging as significant breakthroughs in lightweight aerospace insulation materials. By precisely controlling the microstructure and layering of these materials, researchers are facilitating stepwise thermal insulation across varying temperature gradients, effectively reducing heat transfer rates and pathways. Furthermore, the incorporation of nanotechnology enhances the surface area and creates complex pore structures within insulation materials, further decreasing thermal conductivity and improving overall insulation performance.

Looking ahead, high-temperature lightweight thermal insulation materials for aerospace applications will continue to evolve toward even lighter, thinner, and more resilient solutions capable of withstanding extreme environments. For example, structural modifications of aerogel insulation materials can be achieved through the design approach of thermal insulation tile composites, enhancing both their mechanical and thermal insulation properties. Alternatively, coupling aerogels with multilayer insulation materials can further improve the material’s thermal insulation capacity at high temperatures. With advancements in aerogels, nanofibers, and multilayer composite technologies, these materials will play an increasingly vital role in deep space exploration, hypersonic vehicles, and next-generation space stations. Their ongoing development will ensure stable spacecraft operation in harsh conditions, thereby enhancing humanity’s capabilities for space exploration to new heights.

## Figures and Tables

**Table 2:** Parameters of the Thermal Insulation Tiles [52,53,54,55,56].

Materials	Density (g·cm^3^)	Tensile Strength XY-Direction/MPa Z-Direction/MPa	Thermal Conductivity/W·m^−1^·K^−1^
LI-900	0.144	0.47 0.17	0.036
LI-2200	0.352	1.27 0.53	0.052
FRCI-12	0.192	1.81 0.57	0.046
AETB-8	0.128	0.38 0.69	0.064
BRI-18	0.288	0.96 0.41	0.03

## Data Availability

No new data were created or analyzed in this study.
